# Endobronchial lipoma with tracheobronchial aspergillosis: A case report

**DOI:** 10.1097/MD.0000000000024381

**Published:** 2021-03-19

**Authors:** Yuanyuan Wang, Yongliang Teng, Jie Li, Tingting Lin, Na Lu, Ye Yuan

**Affiliations:** a The Department of Anesthesiology; b The Department of Pathology; c The Department of Pediatrics, The First Hospital of Jilin University, Changchun, China.

**Keywords:** aspergillus, bronchial neoplasm, hemoptysis, lipoma, pneumonectomy

## Abstract

**Introduction:**

Benign neoplasm of the endobronchial tree is quite rare, while endobronchial lipoma is extremely rare. Tracheobronchial aspergillosis is a relatively uncommon but severe form of invasive aspergillosis involving the tracheobronchial tree.

**Patient concerns:**

A 54-year-old male presented to our hospital for investigation and treatment of a cough and hemoptysis.

**Diagnosis:**

The diagnosis was confirmed as endobronchial lipoma with tracheobronchial aspergillosis.

**Interventions:**

The patient received pneumonectomy and voriconazole treatment.

**Outcomes:**

The patient's postoperative course was uneventful, and he was discharged 10 days after surgery. The patient had no evidence of the fungal infection and recurrence during 1 year of follow-up.

**Conclusion:**

Endobronchial lipoma is a rare benign lung tumor, and this is the first report of endobronchial lipoma with tracheobronchial aspergillosis. In patients with suspected endobronchial lipoma, especially those who present with hemoptysis as the initial symptom, it is advisable to exclude coexistent aspergillosis.

## 1 Introduction

Lipomas are common tumors of the body; however, endobronchial lipomas are extremely rare and account for only 0.1–0.5% of all tumors of the lung.^[1]^ They usually grow slowly without specific symptoms and elude detection for months or years. The late diagnosis of this benign neoplasm can lead to severe parenchymal damage owing to bronchial obstruction and subsequent pneumonia.^[2]^ Tracheobronchial aspergillosis is an unusual form of invasive aspergillosis that is primarily limited to the large airways;^[3]^ it can remain asymptomatic or present with hemoptysis, which can be life-threatening. We herein report a case of endobronchial lipoma with severe irreversible pulmonary damage and endobronchial aspergillosis. To the best of our knowledge, this is the first case of endobronchial lipoma with tracheobronchial aspergillosis.

## 2 Case report

**Figure 1:**
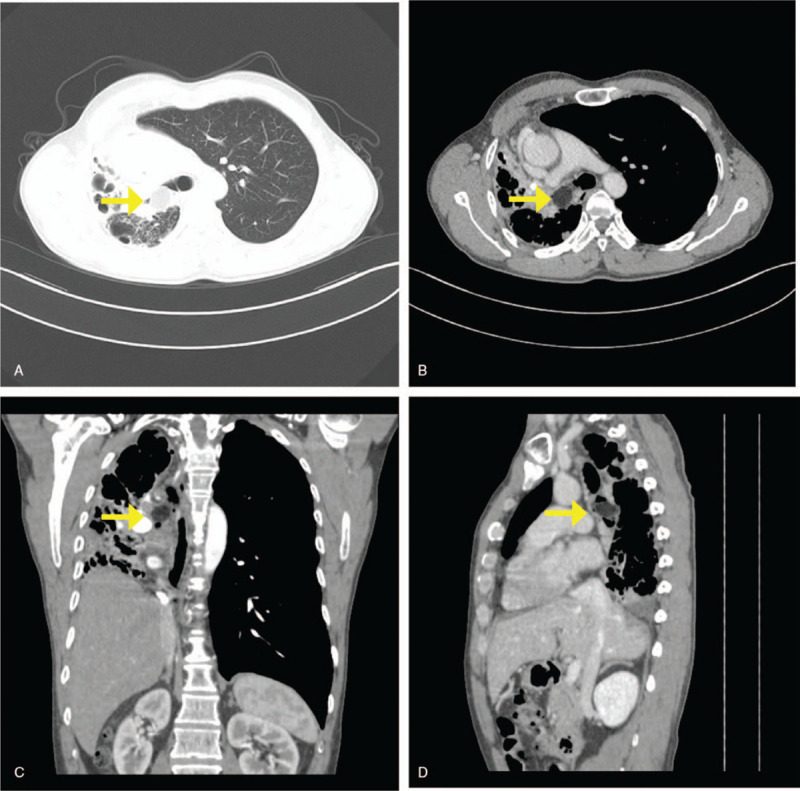
Chest CT showing an extra-bronchial extension of tumor with low attenuation suggesting a fat-containing lesion (arrow). There is an obstruction of the right bronchus intermedius causing complete atelectasis of the right lung with encysted pleurisy and mediastinal shift toward the right; multiple cystic cavities, bronchiectasis, and obstructive pneumonia complicated by pleural effusion could be seen in the right lung.

A 54-year-old man presented with complaints of intermittent cough with episodes of hemoptysis for 1 month. He denied a loss of appetite, weight loss, fevers, chills, or night sweats. He had smoked 40 cigarettes per day for 30 years and had no special past history or medication history. On physical examination, the heart and left lung were normal, but the right hemithorax was dull to percussion with markedly decreased breath sounds. Blood investigations revealed elevated carbon dioxide combining power, C-reactive protein, and erythrocyte sedimentation rate. Two sputum smear examinations for acid-fast bacilli and antibodies against tuberculosis were negative. Blood testing in the patient for HIV-1 and HIV-2 antibodies was negative. Computed tomography (CT) revealed an oval-shaped fat density lesion at the origin of the right main bronchus, 23 × 18 mm in size, with a CT value of −110 HU, causing complete atelectasis of the right lung with encysted pleurisy and mediastinal shift toward the right; multiple cystic cavities, bronchiectasis, and obstructive pneumonia complicated by pleural effusion could be seen in the right lung. CT of the left lung showed coarse lung markings, intrapulmonary puncta and patchy shadows in the upper lobe, and calcification in the lower lobe and lingual lobe (Fig. 1). Flexible bronchoscopy revealed an occluded right main bronchus that was completely occupied by a smooth, yellowish, and polypoid tumor.

**Figure 2:**
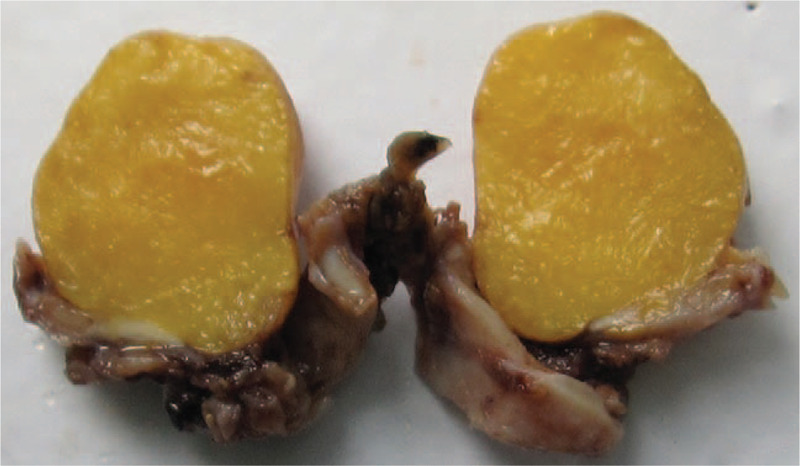
Macroscopic features of the tumor showed a well-circumscribed solid tumor measuring 2.6 × 2.1 × 1.6 cm in maximum dimension. CT: computed tomography.

**Figure 3:**
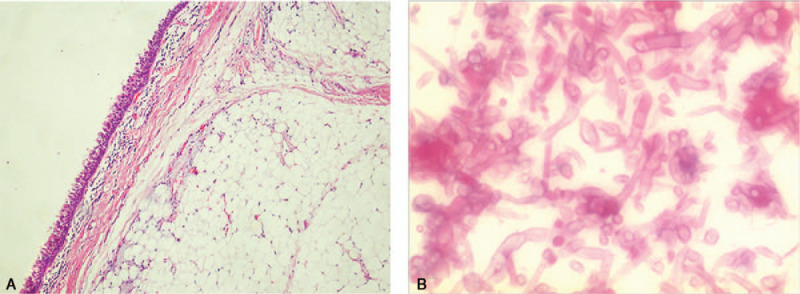
Photomicrograph showing fat tissue surrounded by respiratory epithelium and the submucosa. (A) There are univacuolar mature fat cells, uniform in form and size (H&E, ×100). (B) High-power magnification revealed acutely branching and septate hyphae (H&E, ×100).

Right posterolateral thoracotomy was performed, and at bronchotomy, a yellow, smooth, and fatty lesion was found occluding the right main bronchus, and a frozen section confirmed no neoplastic tissue (Fig. 2). Because of the irreversible damage to the right lung, pneumonectomy was performed. Final pathological examination of the endobronchial mass showed mature adipose tissue growing in the submucosal layer consistent with lipoma, which was not invaded by the fungal infection. (Fig. 3 A). The resected lung specimens also showed bronchiectasis, foreign body granuloma in the right lobe bronchial wall, and fungal infection in the bronchi due to aspergillosis (Fig. 3B). The patient received voriconazole treatment and responded positively. His postoperative course was uneventful, and he was discharged 10 days after surgery. The patient had no evidence of the fungal infection and recurrence during 1 year of follow-up.

## 3 Discussion

Lipoma is a benign mesenchymal tumor composed exclusively of mature adipose tissue. The most common anatomic sites are subcutaneously or under the investing fascia in intramuscular or intermuscular regions, while extremely rare in the trachea with an incidence reported to range from only 0.1% to 0.5% in all lung tumors.^[4]^ Endobronchial lipomas occur more commonly in males in the 4th decade of life or older.^[1]^ The majority are located in the first 3 subdivisions of the tracheobronchial tree with a preference for the right lung ^[5]^ – as in our present case – in which the tumor was at the origin of the right main bronchus.

Symptoms of endobronchial lipomas mainly depend on the degree of airway obstruction. They often grow quietly until they occlude 50–75% of the luminal diameter before cough, stridor, wheezing, and dyspnea on exertion develop.^[6,7]^ Hemoptysis is uncommon because of the avascular nature of lipomas. However, hemoptysis and cough are the most common symptoms of endobronchial aspergilloma, and hemoptysis specifically may be the result of Aspergillus colonization.^[8]^ Our patient was diagnosed with lipoma with aspergillosis, and the symptoms were cough and hemoptysis. The hemoptysis may be due to the impingement of adjacent vascular structures by the lipomas, result from aspergillosis or be due to bronchiectasis. Since most endobronchial lipomas are found after the onset of symptoms, these tumors are often diagnosed late. A delayed diagnosis leads to late treatment, postobstructive pneumonia can occur and result in chronic inflammation and the destruction of the distal lung.^[9]^

CT is valuable in establishing the origin and extent of the tumor. It typically shows an intrabronchial lesion of fat attenuation (−70 UH to −140 UH) with no contrast enhancement. ^[10]^ The most reliable methods for diagnosis are obtained by bronchoscopy and endobronchial biopsy. Bronchoscopic examination typically reveals a yellow polypoid mass with a smooth, regular, and soft surface with little to no vascularization.^[11]^ Endoscopic resection is usually the first line of treatment, and aggressive surgical resection may be indicated if there is permanent distal damage, in the case of recurrence, in the presence of extrabronchial growth or subpleural lipomatous disease, or if technical difficulties during the bronchoscopic procedure are expected.^[12,13]^ The prognosis is usually excellent in completely resected lesions.

Tracheobronchial aspergillosis is a rare form of invasive aspergillosis that is primarily seen in immunocompromised patients or those with local dysfunction of airway immunity,^[3]^ such as carcinomatous cavities of the lung or hematological malignancies, acquired immune deficiency syndrome, a posttransplantation status, or other forms of immunodeficiency. Endobronchial aspergillosis is known to colonize coexisting endobronchial malignancies, especially primary lung malignancies.^[14–16]^ Nevertheless, few cases have been reported in detail in immunocompetent persons suffering from noncavitary lung tumors complicated by aspergillosis, and there have been no reports of endobronchial lipomas with coexisting aspergillosis.

Mycobacterial infection, both tuberculous and nontuberculous, has been identified as the most common primary underlying condition of pulmonary aspergillosis.^[17]^ In addition, a majority of these patients with aspergillosis had risk factors for chronic lung disease, most commonly long-term smoking.^[15]^ However, there is no strong correlation between aspergillosis and neoplasm development either in the literature or in this article. The coexistence of the endobronchial lipoma in the last setting could well be either coincidental or secondary to mutual risk factors.

The radiologic findings of tracheobronchial aspergillosis are unspecific and elusive, such as thickening of the trachea or bronchial wall, atelectasis, or obstructive pneumonia caused by stenosis of the lumen and poor drainage, and coexistence of multiple multi lobar lung exudates, consolidation, and cavities.^[18]^ The diagnosis of tracheobronchial aspergillosis depends on histopathological examination, so bronchoscopy biopsy is of great significance. Voriconazole is the recommended therapy for patients with invasive aspergillosis. The clinical course and prognosis largely depend on the host underlying disease.^[18]^

## 4 Conclusion

Endobronchial lipoma is a rare benign lung tumor of which clinicians should be aware, and early resection is essential to avoid permanent pulmonary damage. In patients with suspected endobronchial lipoma, especially those who present with hemoptysis as the initial symptom, even if radiology or bronchoscopy is suggestive of the diagnosis, it is advisable to exclude coexistent aspergillosis.

## Author contributions

**Approval of article:** Yuanyuan Wang, Yongliang Teng, Jie Li, Tingting Lin, Na Lu, Ye Yuan

**Concept/design:** Yuanyuan Wang, Ye Yuan.

**Critical revision of article:** Yuanyuan Wang, Yongliang Teng, Jie Li, Tingting Lin, Na Lu, Ye Yuan

**Conceptualization:** Yong-Liang Teng, Ting-Ting Lin, Na Lu, Ye Yuan.

**Investigation:** Yuan-Yuan Wang, Yong-Liang Teng, Jie Li, Ting-Ting Lin, Na Lu.

**Methodology:** Yuan-Yuan Wang, Ye Yuan.

**Resources:** Ye Yuan.

**Supervision:** Na Lu.

**Validation:** Na Lu.

**Writing – original draft:** Yuan-Yuan Wang, Jie Li, Ting-Ting Lin, Na Lu.

**Writing – review & editing:** Na Lu, Ye Yuan.
